# Hospitalizations due to stroke and their relationship with Chagas disease: a population-based analytical study using data from the Hospital Information System of the Brazilian National Health System, Minas Gerais, Brazil, 2022

**DOI:** 10.1590/S2237-96222026v35e20240901.en

**Published:** 2025-11-21

**Authors:** Ana Clara de Jesus Santos, Nayara Dornela Quintino, Jeniffer Eduarda Firmino Almeida Silva, Maria do Carmo Pereira Nunes, João Alves Pereira, Ester Cerdeira Sabino, Ariela Ferreira Mota, Desirée Sant'Ana Haikal

**Affiliations:** 1 Universidade Estadual de Montes Claros, Programa de Pós-Graduação em Ciências da Saúde, Montes Claros, MG, Brazil; 2 Secretaria de Estado de Saúde de Minas Gerais, Unidade Regional de Saúde de Divinópolis, Divinópolis, MG, Brazil; 3 Universidade Federal de Minas Gerais, Departamento de Medicina Interna, Belo Horizonte, MG, Brazil; 4 Universidade de São Paulo, Faculdade de Medicina, São Paulo, SP, Brazil; 5 Secretaria de Estado de Saúde de Minas Gerais, Montes Claros, MG, Brazil; 6 Universidade Estadual de Montes Claros, Departamento de Enfermagem, Montes Claros, MG, Brazil

**Keywords:** Chagas Disease, Stroke, Hospitalization, Hospital Information Systems, Secondary Data Analysis, Enfermedad de Chagas, Accidente Cerebrovascular, Hospitalización, Sistemas de Información en Hospital, Análisis de Datos Secundarios

## Abstract

**Objective:**

To assess whether hospitalizations due to stroke were associated with Chagas disease and the Chagas disease vulnerability index in the state of Minas Gerais.

**Methods:**

This was a population-based analytical observational study. Data were obtained from the Hospital Information System of the Brazilian National Health System in Minas Gerais for the year 2022. Hospitalization records were selected based on the International Statistical Classification of Diseases and Related Health Problems, in which stroke was recorded as the primary cause and Chagas disease/sentinels as the secondary cause. Stroke-related hospitalization rates were estimated, and a correlation analysis was conducted between the Chagas disease vulnerability index and stroke hospitalization prevalence in Minas Gerais and its health macro-regions.

**Results:**

In 2022, a total of 25,813 hospitalizations due to stroke as the primary cause were recorded, of which only 18 included Chagas disease/sentinels as the secondary cause, most of them in the Central macro-region (n=10). The correlation between stroke-related hospitalizations and the Chagas disease vulnerability index was weak (r=0.145) and not statistically significant, despite the overlap of macro-regions with higher stroke-related hospitalization rates and higher Chagas disease vulnerability index scores (Jequitinhonha Valley and North).

**Conclusion:**

Although there was a high number of stroke-related hospitalizations in the Brazilian National Health System in Minas Gerais, only 18 of these records were associated with Chagas disease/sentinels, which may suggest underreporting of Chagas disease and uncertainty regarding the quality of such records.

## Resumen

**Objetivo:**

Evaluar si las hospitalizaciones por Accidente Cerebrovascular (ACV) presentaron relación con la enfermedad de Chagas y con el índice de vulnerabilidad de la enfermedad de Chagas en Minas Gerais.

**Métodos:**

Se trata de un estudio observacional analítico de base poblacional. Se utilizaron registros del Sistema de Información Hospitalaria del Sistema Único de Saúde de Minas Gerais de 2022. La selección de los datos de hospitalizaciones se basó en la Clasificación Estadística Internacional de Enfermedades y Problemas Relacionados con la Salud, en la que el ACV se registró como causa primaria y la enfermedad de Chagas/sentinelas, como causa secundaria. Se estimaron las tasas de hospitalizaciones por ACV y se realizó un análisis de correlación entre el índice de vulnerabilidad de la enfermedad de Chagas y la prevalencia de hospitalizaciones por ACV para Minas Gerais y sus macrorregiones.

**Resultados:**

En 2022, se registraron 25 813 hospitalizaciones por ACV como causa primaria y, de estas, solo 18 presentaban un registro de la enfermedad de Chagas/sentinelas como causa secundaria, la mayoría de ellos registrados en la macrorregión Centro (n=10). La correlación entre las hospitalizaciones por ACV y el índice de vulnerabilidad a la enfermedad de Chagas fue débil (r=0,145) y no significativa, a pesar de la coincidencia entre las macrorregiones con mayores tasas de hospitalización por ACV y mayores índices de vulnerabilidad a la enfermedad de Chagas (Vale do Jequitinhonha y Norte).

**Conclusione:**

s: Aunque hay un alto número de hospitalizaciones por ACV en el Sistema Único de Salud en Minas Gerais, solo 18 de estos registros se relacionaron con la enfermedad de Chagas/sentinelas, lo que puede sugerir un subregistro relativo a la enfermedad de Chagas e incertidumbre sobre la calidad de dichos registros.

**Ethical aspects**

This research used public domain anonymized databases.

## Introduction 

Chagas disease is a potentially lethal illness caused by the protozoan *Trypanosoma cruzi*. It is one of a group of neglected tropical diseases (1,2). An estimated 20.0% to 30.0% of individuals with Chagas disease develop the chronic form of cardiomyopathy associated with the disease, which is characterized by a wide range of clinical manifestations (3). Among these manifestations, the most notable are life-threatening arrhythmias, heart failure, and thromboembolic events, with ischemic stroke being one of the most relevant (3).

Chronic Chagas cardiomyopathy is independently associated with ischemic stroke (4-7). The risk of death from stroke among individuals with Chagas disease may reach 30.0%, which is twice as high as in uninfected individuals, with an estimated stroke mortality rate of 4.6 per 1,000 patients per year (8). An incidence rate of 12.2 strokes per 1,000 patients per year has been reported, although the study did not include a control group without Chagas disease (9). In a cohort of patients with heart failure, Chagas disease was found to be a predictor of stroke and death, regardless of the severity of heart disease, and the incidence of stroke was higher among patients with Chagas disease compared to those without the disease (20.2 versus 13.9 events per 1,000 patients per year) (4).

Brazil has several endemic regions for Chagas disease, and the state of Minas Gerais stands out as one of the states with the highest prevalence rates (10). Although the relationship between stroke and Chagas disease has been demonstrated by robust previous studies (4-7), it is believed that Chagas disease remains underreported even in endemic areas. It is also believed that many stroke cases resulting from Chagas disease may present to health services without this connection being identified due to the lack of serological testing for Chagas disease. 

This study aimed to assess, based on hospitalization records from the Hospital Information System of the Brazilian National Health System (SIH/SUS) in Minas Gerais and its macro-regions, whether hospitalizations due to stroke showed any relationship with Chagas disease and with the Chagas disease vulnerability index in 2022.

## Methods 

### Study Design

This was a population-based analytical observational study that used secondary data from the SIH/SUS. It is an administrative registry system of the Information Technology Department of the Brazilian National Health System (DATASUS), which records all hospitalizations covered by SUS. It is a publicly available database maintained by the Brazilian Ministry of Health. 

### Setting 

The study used SIH/SUS records from the state of Minas Gerais and its respective health macro-regions for the year 2022. Minas Gerais has an estimated population of 20,539,989 inhabitants, a Human Development Index of 0.774, and is divided into 14 health macro-regions (Central, Central-South, Jequitinhonha, East, Southeast, Northeast, Northwest, North, West, South, Triângulo do Norte, Triângulo do Sul, Vale do Aço). Data were extracted in November 2023.

### Participants 

Hospitalization records were selected for individuals residing in Minas Gerais whose primary cause of admission was stroke. The following ICD-10 ￼codes were used: I63 – Cerebral infarction and I64 – Stroke not specified as hemorrhagic or infarction (11), as specified in the International Statistical Classification of Diseases and Related Health Problems 10th Revision (ICD-10). Records were excluded if stroke was not listed as the primary diagnosis, if the patient was hospitalized in Minas Gerais but resided in another state, or if the “identification=5” field was present, which referred to long-stay hospitalizations in psychiatric specialties, prolonged care, or home hospitalization.

### Variables 

￼Among the hospitalizations that listed stroke as the primary cause, Chagas disease (or its indicators) was investigated as a secondary cause, also based on ICD-10 codes. The following codes were used: B57.0 – Acute Chagas disease with heart involvement; B57.1 – Acute Chagas disease without heart involvement; B57.2 – Chagas disease (chronic) with heart involvement; B57.3 – Chagas disease (chronic) with digestive system involvement; B57.4 – Chagas disease (chronic) with nervous system involvement; B57.5 – Chagas disease (chronic) with other organ involvement; K93.1 – Megacolon in Chagas disease; K23.1 – Megaoesophagus in Chagas disease (11). 

Sentinel conditions were also considered—these are indicators related to diseases or conditions resulting from the progression of chronic Chagas disease, as defined in the Chagas Disease Vulnerability Bulletin (12). The following sentinel ICD-10 codes were adopted: I50.0 – Heart failure; I42.0 – Dilated cardiomyopathy; I42.9 – Cardiomyopathy, unspecified; I44 – Atrioventricular and left bundle-branch block (all subcategories from 0 to 7); I46.1 – Cardiac arrest; I49.9 – Cardiac arrhythmia, unspecified; I51.7 – Cardiomegaly (12). 

To characterize individuals hospitalized due to stroke and diagnosed secondarily with Chagas disease/sentinel conditions, the following variables were evaluated: sex, age, race/skin color, city, macro-region of residence, and occurrence of death. Additional data were collected from other public databases. 

Population data for Minas Gerais and its macro-regions were obtained from the 2022 Demographic Census of the Brazilian Institute of Geography and Statistics (IBGE) (13). They were used to estimate stroke-related hospitalization rates. 

Data on the Chagas Disease Vulnerability Index for the state and its macro-regions were also collected. This index, proposed by the Brazilian Ministry of Health in 2022, aims to identify areas with a higher potential for morbidity and mortality from chronic Chagas disease, considering barriers to access to health services, low suspicion and detection of chronic cases, and impacts on quality of life for people affected by the disease (12). The Vulnerability index for Chagas disease comprises three sub-indices: (i) epidemiological indicators directly related to chronic Chagas disease; (ii) indicators related to diseases/conditions resulting from disease progression; and (iii) indicators related to access to health services (12).

### Data sources

Data were obtained from the SIH/SUS, the 2022 Demographic Census conducted by the Brazilian Institute of Geography and Statistics, and the Chagas Disease Vulnerability Index. 

### Bias 

The use of secondary data may have introduced registration bias and underreporting.

### Study Size 

The study included 25,813 hospitalization records for stroke in Minas Gerais. The data refer exclusively to patients hospitalized in beds accredited by SUS.

### Statistical methods

Crude and standardized rates (per 100,000 inhabitants) of stroke-related hospital admissions covered by SUS were estimated. These data were presented in a map. Chagas disease vulnerability index data for Minas Gerais and its macro-regions were presented as continuous numerical values. To test the association between the standardized stroke-related hospitalization rate and the Chagas disease vulnerability index, Spearman’s correlation test was applied. Subsequently, the number and characteristics of individuals hospitalized for stroke (primary diagnosis) and Chagas disease/sentinel conditions (secondary diagnosis) were estimated. 

SIH/SUS data were downloaded in “.dbc” format, merged, and saved as “.dbf” using TabWin 4.15 software (14). Stata version 17 (StataCorp, College Station, Texas, United States) and SPSS version 20.0 (Statistical Package for the Social Sciences, IBM) were used for database management (15), record selection, and statistical analyses.

## Results 

**Figure 1:**
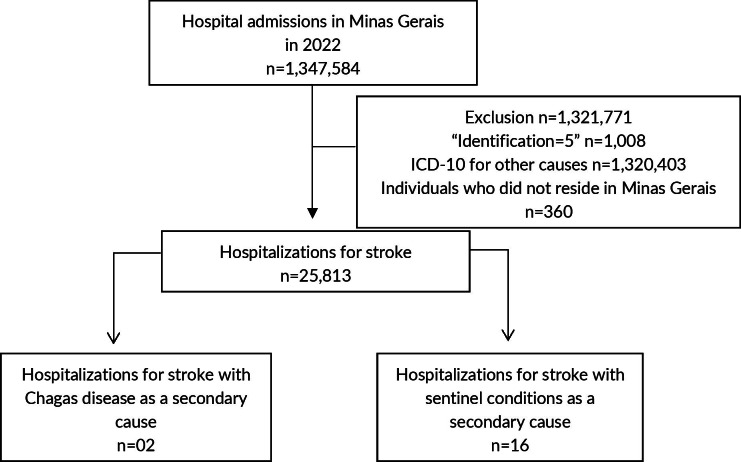
Number of hospital admissions via the hospital information system and selection of admissions of interest, according to the inclusion and exclusion criteria adopted in this study. Minas Gerais, 2022 (n=1,347,584)

A total of 1,347,584 hospitalizations were recorded via SIH/SUS in Minas Gerais in 2022. Of these, 1,321,771 were excluded based on the study’s exclusion criteria (Figure 1).

Among the stroke-related hospitalizations covered by SUS in Minas Gerais (n=25,823), 52.2% occurred among males, ranging from 49.8% to 56.6% across macro-regions. The mean age was 67.4 years, ranging from 66.8 to 70.6 years by macro-region. Most individuals self-identified as mixed race/skin color (43.5%) and did not die during hospitalization (87.2%).

**Figure 2:**
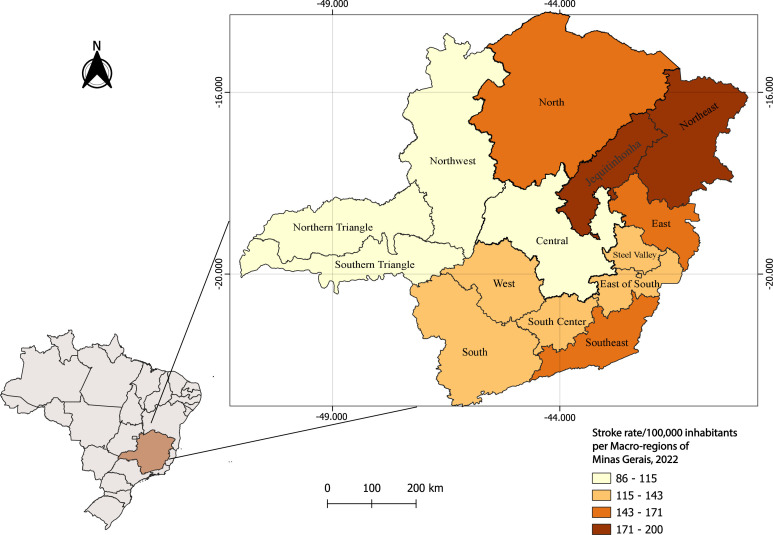
Hospitalization rates for stroke. Macro-regions of Minas Gerais, 2022

The overall stroke-related hospitalization rate in Minas Gerais was 125.7 per 100,000 inhabitants. Among the state’s macro-regions, Vale do Jequitinhonha, Northeast, and North had the highest rates: 199.7, 177.2, and 166.3 per 100,000 inhabitants, respectively (Figure 2). The highest Chagas disease vulnerability index scores were found in Vale do Jequitinhonha, North, and Northwest regions of Minas Gerais (Table 1).

**Table 1:** Calculated hospitalization rate for stroke and Chagas disease vulnerability index. Macro-regions of Minas Gerais, 2022 (n=25,813)

Macro-regions	n (%)	Population	Hospitalization rate for stroke/100,000 inhabitants	Vulnerability index for Chagas disease
Central	6,944 (26.9)	6,376,699	108.9	0.154
Central-South	1,062 (4.1)	765,591	138.7	0.214
Vale do Jequitinhonha	770 (3.0)	385,590	199.7	0.593
East	920 (3.6)	643,031	143.1	0.154
Southeast	949 (3.7)	690,204	137.5	0.125
Northeast	1,373 (5.3)	774,768	177.2	0.203
Northwest	615 (2.4)	712,909	86.3	0.494
North	2,682 (10.4)	1,612,749	166.3	0.550
West	1,632 (6.3)	1,288,118	126.7	0.190
Southeast	2,511 (9.7)	1,596,341	157.3	0.216
South	3,473 (13.5)	2,796,445	124.2	0.197
Triângulo do Norte	1,169 (4.5)	1,318,243	88.7	0.333
Triângulo do Sul	766 (3.0)	787,105	97.3	0.427
Vale do Aço	947 (3.7)	790,925	119.7	0.117

**Figure 3:**
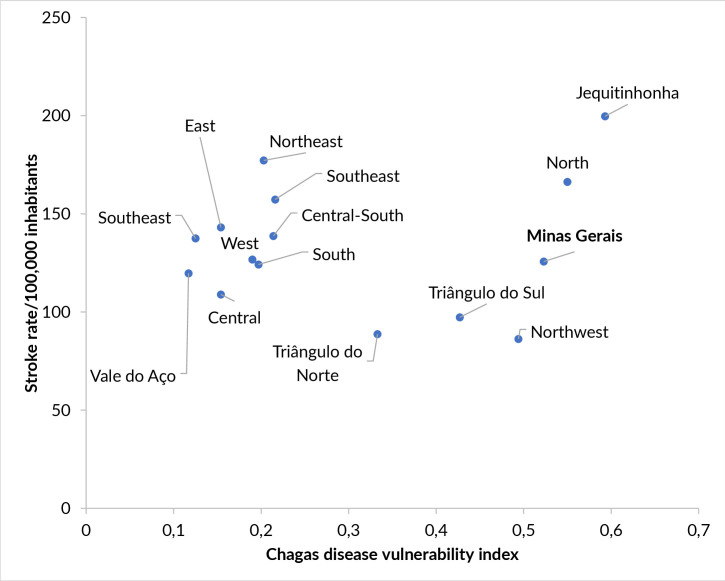
Relationship between the hospitalization rate for stroke per 100,000 inhabitants and the Chagas disease vulnerability index. Macro-regions of Minas Gerais, 2022

The correlation between the stroke-related hospitalization rate and the Chagas disease vulnerability index was weak (r=0.145; p-value 0.607), although macro-regions such as Vale do Jequitinhonha and North showed both high stroke-related hospitalization rates and high vulnerability index scores (Figure 3).

Of all stroke-related hospitalizations in the state, two were recorded with Chagas disease as the secondary cause, and 16 were associated with sentinel conditions for Chagas disease (Figure 1). Among these 18 cases, 12 were women, only three individuals were aged 60 or younger, nine were of Brown, five died following stroke, and all deaths occurred among females. Ten of the 18 cases were recorded in the Central macro-region (Table 2).

**Table 2:** Description of cases of hospitalizations for stroke as the primary cause and Chagas disease/sentinel lesions as the secondary cause. Minas Gerais, 2022 (n=18)

Hospitalizations for stroke with Chagas disease as secondary cause	Primary ICD-10^a^	Secondary ICD-10	Sex	Age	Race/skin color	City	Macro-region	Death
1st	I64	B57.2^b^	Male	59	Black	Pouso Alegre	South	No
2nd	I64	B57^c^	Female	82	Black	Uberlândia	Triângulo do Norte	No
3rd	163.9	144.2	Female	96	No information	Belo Horizonte	Central	Yes
4th	I64	I50.0	Female	83	Asian	Belo Horizonte	Central	Yes
5th	I64	I50.0	Male	52	Black	Belo Horizonte	Central	No
6th	I64	I50.0	Female	75	White	Uberlândia	Triângulo do Norte	No
7th	I63.4	I42.9	Female	82	White	Tocos do Moji	South	Yes
8th	I64	I50.0	Male	59	Brown	Sabinópolis	Central	No
9th	I64	I50.0	Female	60	Brown	Sabinópolis	Central	No
10th	I64	I50.0	Male	64	Brown	Sabinópolis	Central	No
11th	I64	I50.0	Male	73	Black	Sabinópolis	Central	No
12th	I64	I50.0	Female	62	Brown	Sabinópolis	Central	No
13th	I64	I49.9	Male	61	White	Pouso Alegre	South	No
14th	I64	I42.9	Female	71	White	Monte Sião	South	No
15th	I64	I50.0	Female	95	Brown	Materlândia	Central	No
16th	I64	I49.9	Female	83	Brown	Juatuba	Central	Yes
17th	I64	I44	Female	78	White	Cambuí	South	No
18th	I64	I46.1	Female	62	White	Bom Despacho	West	Yes

^a^ICD-10: International Statistical Classification of Diseases and Related Health Problems 10th Revision; ^b^Specific secondary ICD-10 code for chronic Chagas disease with heart involvement; ^c^Specific secondary ICD-10 code for Chagas disease.

## Discussion 

This study identified a high rate of stroke-related hospitalizations covered by SUS in Minas Gerais in 2022. These rates varied considerably across the state’s macro-regions. Macro-regions with higher stroke-related hospitalization rates also exhibited the highest Chagas disease vulnerability index scores; however, the correlation between stroke-related hospitalization rates and Chagas disease vulnerability index scores was weak. Of all stroke-related hospitalizations in the state, only 18 were recorded with Chagas disease/sentinel conditions as the secondary cause, most of which were in the Central macro-region. These findings suggest underreporting of Chagas disease.

These data must be interpreted in light of the study’s limitations. It is based on secondary data, which always carries uncertainty regarding data quality. Although a public and robust database was used, SIH/SUS records are primarily intended for hospital billing purposes (16); they are not specifically designed for research, which may result in inconsistent and non-standardized data, as well as underreporting of records. 

Despite its limitations, in recent years, there has been growing interest among the scientific community in the use of large-scale secondary databases from health services (17,18) due to their broad population coverage and the opportunity to explore large samples at a low cost (18-20). Therefore, the results presented here are relevant, as they encompass hospitalizations in SUS-accredited beds across all of Minas Gerais, thereby adding value and interpretability to existing records (18-20). 

The macro-regions of Minas Gerais with the highest rates of stroke-related hospitalizations covered by SUS corresponded to areas with the highest Chagas disease vulnerability index scores in Brazil—namely, Vale do Jequitinhonha and the North. This finding supports the hypothesis of a possible relationship between stroke and Chagas disease. However, no records of stroke as the primary cause and Chagas disease/sentinel conditions as the secondary cause were found in the aforementioned macro-regions, which are known to be hyperendemic for the disease (21,12). It was an unexpected finding, as these regions had the highest endemicity for Chagas disease (9.2%) (21). These regions also have a known history of precarious healthcare access for Chagas disease patients (22,23), where access to adequate medical care remains a significant challenge for the health system. Shortcomings in healthcare services related to Chagas disease in these regions may explain the low number of records, which likely do not reflect reality (22). 

The Central macro-region, where the state capital is located, demonstrated better development and more organized healthcare services (24,25). Although not hyperendemic for Chagas disease, it had the highest number of hospitalizations recorded with stroke as the primary diagnosis and Chagas disease/sentinel conditions as secondary. This finding reinforces the hypothesis that the organization of healthcare services may have a more substantial influence on the quality of hospitalization records than the epidemiological context itself. An assessment of hospital service performance by level of care conducted in 2010 showed that the Central macro-region had a resolvability of over 95.0%, while the Vale do Jequitinhonha macro-region had a resolvability of only 28.0%. Resolution capacity below 30.0% indicates care gaps (25).

Among stroke-related hospitalizations covered by SUS in Minas Gerais, only two cases had Chagas disease recorded as a secondary diagnosis, and 16 others had sentinel conditions. The findings suggest that Chagas disease may be underreported in hospital records. The Brazilian Ministry of Health acknowledged the underreporting of Chagas disease, noting a prevalence of 2.1% in Minas Gerais and recognizing that the number of reported patients was far below the actual number of people with the disease (26). If Chagas disease diagnoses are underreported in general, it is unsurprising that this underreporting would be even greater when obscured by an emergency condition such as stroke. Chagas disease is a neglected disease (12), both by patients—who may not inform health professionals of their diagnosis—and by professionals—who may not associate the complications of Chagas disease with the disease itself (22,23).

The limited number of stroke records with Chagas disease as a secondary diagnosis makes it difficult to accurately estimate the magnitude of the association between these two conditions within the public hospital context. A higher number of records was expected, given that several studies in the literature have identified a significant relationship between Chagas disease and the occurrence of stroke (4,27). A total of 201 participants were assessed, including 100 controls (without stroke) and 101 cases (with stroke). After performing serology for Chagas disease, it was observed that, in the control group, only two individuals tested positive, while in the case group, 14 individuals tested positive for Chagas disease. A positive serology result (OR 7.17; 95%CI 1.50; 34.19) was independently associated with stroke (27).

A previous incidence of stroke was found to be 20.2 per 1,000 among people with Chagas disease and 13.9 per 1,000 among people without Chagas disease (p-value 0.48). Chagas disease was an independent predictor of death (41.6 vs. 43.1 per 1,000 among people with Chagas disease and without Chagas disease) (4). In a cardiology outpatient clinic in Bahia, ischemic stroke was significantly more frequent among patients with Chagas disease than among those with non-Chagas embolic cardiomyopathies (15.0% vs. 6.3%; p-value 0.01) (6). Pathological studies of the brain in patients with chronic Chagas disease have reported cerebral infarction in 9.4% to 36.0% of cases (28). Additionally, the diagnosis of Chagas disease may be established following a stroke in up to 40.0% of cases (5). It has been suggested that the occurrence of stroke could provide an opportunity for diagnosing Chagas disease (27.5%). Conversely, patients with Chagas disease should be informed of their increased stroke risk and properly monitored (5). All these studies demonstrate the relationship between Chagas disease and stroke and support the hypothesis that a portion of stroke-related hospitalizations may involve undiagnosed Chagas disease, especially in hyperendemic areas.

The stroke-related hospitalization rate is an indirect indicator of the availability of basic preventive and control measures for hypertensive disease. It is helpful in planning, managing, and evaluating adult healthcare policies and actions (29). Just as stroke is associated with hypertension and other health conditions (30), scientific literature has already indicated a higher stroke risk among individuals with Chagas disease (4), though this association remains underrecognized. Stroke cases should represent an opportunity to diagnose Chagas disease and help reduce the invisibility surrounding it.

In 2022, there was a high prevalence of stroke-related hospitalizations in Minas Gerais covered by SUS, particularly in the Vale do Jequitinhonha and North macro-regions—areas characterized by a high Chagas disease vulnerability index and recognized as hyperendemic for this infection. However, only 18 cases were recorded, with stroke as the primary cause and Chagas disease/sentinel conditions as the secondary cause. None of these were recorded in the hyperendemic macro-regions, with most observed in the Central macro-region. 

These findings underscore the need to raise awareness among health professionals about the systematic investigation of Chagas disease in patients hospitalized for stroke in order to improve diagnosis, notification, and follow-up, as well as ensure the greater quality and reliability of SIH/SUS records. Such an initiative is essential to addressing the historical neglect that still characterizes Chagas disease in Brazil.
