# Supplementary material for: Hospitalizations due to stroke and their relationship with Chagas disease: a population-based analytical study using data from the Hospital Information System of the Brazilian National Health System, Minas Gerais, Brazil, 2022
Source: Epidemiol Serv Saude. 2025 Nov 21;35:e20240901. doi: 10.1590/S2237-96222026v35e20240901.en (PMC12641598; doi:10.1590/S2237-96222026v35e20240901.en)
Supplement: Translation

# Translation: Internações por acidente vascular cerebral e sua relação com a doença de Chagas: estudo analítico de base populacional com dados do Sistema de Informações Hospitalares do Sistema Único de Saúde, Minas Gerais, 2022

**DOI:** 10.1590/S2237-96222026v35e20240901.pt

**Authors:** Ana Clara de Jesus Santos, Nayara Dornela Quintino, Jeniffer Eduarda Firmino Almeida Silva, Maria do Carmo Pereira Nunes, João Alves Pereira, Ester Cerdeira Sabino, Ariela Ferreira Mota, Desirée Sant'Ana Haikal

**Affiliations:** 1 Universidade Estadual de Montes Claros, Programa de Pós-Graduação em Ciências da Saúde, Montes Claros, MG, Brasil; 2 Secretaria de Estado de Saúde de Minas Gerais, Unidade Regional de Saúde de Divinópolis, Divinópolis, MG, Brasil; 3 Universidade Federal de Minas Gerais, Departamento de Medicina Interna, Belo Horizonte, MG, Brasil; 4 Universidade de São Paulo, Faculdade de Medicina, São Paulo, SP, Brasil; 5 Secretaria de Estado de Saúde de Minas Gerais, Montes Claros, MG, Brasil; 6 Universidade Estadual de Montes Claros, Departamento de Enfermagem, Montes Claros, MG, Brasil

**Keywords:** Doença de Chagas, Acidente Vascular Cerebral, Hospitalização, Sistemas de Informação Hospitalar, Análise de Dados Secundários

## Abstract

**Objetivo:**

Avaliar se as internações por acidente vascular cerebral (AVC) apresentaram relação com a doença de Chagas e com o índice de vulnerabilidade da doença de Chagas em Minas Gerais.

**Métodos:**

Tratou-se de estudo observacional analítico de base populacional. Utilizaram-se registros do Sistema de Informações Hospitalares do Sistema Único de Saúde de Minas Gerais de 2022. A seleção dos dados de internações hospitalares foi baseada na Classificação Estatística Internacional de Doenças e Problemas Relacionados com a Saúde, na qual o AVC foi registrado como causa primária e a doença de Chagas/sentinelas, como causa secundária. Taxas de internações por AVC foram estimadas e conduziu-se análise de correlação do índice de vulnerabilidade da doença de Chagas e da prevalência de internações por AVC para Minas Gerais e suas macrorregiões.

**Resultados:**

Em 2022, foram registradas 25.813 internações por AVC como causa primária, e destas, somente 18 apresentaram registro da doença de Chagas/sentinelas como causa secundária, sendo a maioria registrada na macrorregião Centro (n=10). A correlação entre internações por AVC e o índice de vulnerabilidade da doença de Chagas foi fraca (r=0,145) e não significativa, apesar da coincidência entre macrorregiões com maiores taxas de internação por AVC e maiores índices de vulnerabilidade da doença de Chagas (Vale do Jequitinhonha e Norte).

**Conclusões:**

Embora haja alto número de internações por AVC no Sistema Único de Saúde em Minas Gerais, somente 18 desses registros foram relacionados à doença de Chagas/sentinelas, o que pode sugerir subnotificação relativa à doença de Chagas e incerteza quanto à qualidade de tais registros.

**Aspectos éticos**

Esta pesquisa utilizou bancos de dados de domínio público e anonimizados.

## Introdução 

A doença de Chagas é uma enfermidade potencialmente letal, causada pelo protozoário *Trypanosoma cruzi* e faz parte do grupo das doenças tropicais negligenciadas (1,2). Estimou-se que entre 20,0% e 30,0% dos indivíduos com a doença de Chagas evoluam para a forma crônica da cardiomiopatia associada à doença, a qual se caracteriza pela ampla gama de manifestações clínicas (3). Entre essas, destacam-se as arritmias com risco de morte súbita, a insuficiência cardíaca e os eventos tromboembólicos, sendo o acidente vascular cerebral (AVC) isquêmico um dos mais relevantes (3).

A cardiomiopatia crônica da doença de Chagas está independentemente associada ao AVC isquêmico (4-7). O risco de morte por AVC em indivíduos com doença de Chagas pode chegar a 30,0%, que corresponde ao dobro em relação aos indivíduos não infectados, sendo a taxa de mortalidade por AVC estimada em 4,6 por 1 mil pacientes por ano (8). Foi identificada a incidência de AVC de 12,2 por 1 mil pacientes por ano, porém não incluiu um grupo controle sem doença de Chagas (9). Em uma coorte de pacientes com insuficiência cardíaca, a doença de Chagas foi um preditor de AVC e morte independentemente da gravidade da doença cardíaca, e a incidência de AVC foi maior em pacientes com doença de Chagas quando comparados aos sem doença de Chagas (20,2 versus 13,9 eventos por 1 mil pacientes por ano) (4).

O Brasil apresenta diversas regiões endêmicas para a doença de Chagas, e Minas Gerais se destaca como um dos estados com os maiores índices de prevalência (10). Embora a relação entre AVC e doença de Chagas tenha sido verificada por estudos prévios robustos (4-7), acredita-se que a doença de Chagas permanece subnotificada mesmo em regiões endêmicas. Acredita-se também que muitos casos de AVC em decorrência da doença de Chagas podem se apresentar aos serviços de saúde sem, contudo, ter tal relação observada pela falta dos testes sorológicos para a doença de Chagas. 

Neste estudo, objetivou-se avaliar, por meio de registros de internações hospitalares do Sistema de Informações Hospitalares do Sistema Único de Saúde (SIH/SUS) de Minas Gerais e suas macrorregiões, se as internações por AVC apresentaram alguma relação com a doença de Chagas e com o índice de vulnerabilidade da doença de Chagas em 2022.

## Métodos 

### Delineamento do estudo

Tratou-se de estudo observacional analítico de base populacional, que utilizou dados secundários do SIH/SUS. Este é um sistema de registro administrativo do Departamento de Informação e Informática do Sistema Único de Saúde que registra todos os atendimentos hospitalares financiados pelo SUS. Trata-se de base de dados pública processada pelo Ministério da Saúde. 

### Contexto 

O estudo utilizou registros do SIH/SUS de Minas Gerais e suas respectivas macrorregiões referentes a 2022. Minas Gerais possui população estimada de 20.539.989 habitantes, índice de desenvolvimento humano 0,774 e é separada por 14 macrorregiões (Centro, Centro Sul, Jequitinhonha, Leste, Leste do Sul, Nordeste, Noroeste, Norte, Oeste, Sudeste, Sul, Triângulo do Norte, Triângulo do Sul e Vale do Aço). Os dados foram extraídos em novembro de 2023.

### Participantes 

Utilizaram-se registros de indivíduos que residiam em Minas Gerais cuja causa primária da internação foi o AVC. Foram utilizados os seguintes ￼códigos: I63 – Infarto cerebral e I64 – Acidente vascular cerebral não especificado como hemorrágico ou isquêmico (11), conforme especificações da 10ª revisão da Classificação Estatística Internacional de Doenças e Problemas Relacionados com a Saúde (CID-10). Foram excluídos os registros de internações que não constaram AVC no diagnóstico principal, os pacientes internados em hospitais de Minas Gerais que residiam em outro estado e os pacientes com “identificação=5”, que se referiam aos casos de longa permanência do paciente nas especialidades de psiquiatria e pacientes sob cuidados prolongados e internação domiciliar.

### Variáveis 

Entre os registros das internações que indicavam o AVC como causa primária, buscou-se a doença de Chagas (ou seus indícios) como causa secundária, baseado também no CID-10. Foram utilizados os seguintes códigos: B57.0 – Forma aguda da doença de Chagas, com comprometimento cardíaco; B57.1 – Forma aguda da doença de Chagas, sem comprometimento cardíaco; B57.2 – Doença de Chagas (crônica) com comprometimento cardíaco; B57.3 – Doença de Chagas (crônica) com comprometimento do aparelho digestivo; B57.4 – Doença de Chagas (crônica) com comprometimento do sistema nervoso; B57.5 – Doença de Chagas (crônica) com comprometimento de outros órgãos; K93.1 – megacólon na doença de Chagas; K23.1 – megaesôfago na doença de Chagas (11). 

Consideraram-se também condições sentinelas, que são indicadores relacionados às doenças/agravos decorrentes da evolução da doença de Chagas, conforme apresentado no Boletim de Vulnerabilidade da doença de Chagas (12). Como CID-10 sentinelas foram adotados os seguintes códigos: I50.0 – Insuficiência cardíaca; I42.0 – Cardiomiopatia dilatada; I42.9 – Cardiomiopatia não especificada; I44 – Bloqueio atrioventricular (todas as categorias, de 0 a 7); I46.1 – Morte súbita de origem cardíaca; I49.9 – Arritmia cardíaca não especificada; e I51.7 – Cardiomegalia (12). 

Para a caracterização dos participantes internados por AVC e com diagnóstico secundário da doença de Chagas/sentinelas, foram avaliadas as variáveis sexo, idade, raça, cidade, macrorregião de residência e ocorrência de morte. Adicionalmente, dados foram coletados de outras bases de dados públicas. 

Dados de porte populacional do estado e suas macrorregiões foram obtidos do último Censo Demográfico (2022) do Instituto Brasileiro de Geografia e Estatística (13). Tais dados foram utilizados para estimar as taxas de internação por AVC. 

Foram também coletadas informações sobre o índice de vulnerabilidade da doença de Chagas do estado e por macrorregião. Tal índice, proposto pelo Ministério da Saúde em 2022, objetivou demonstrar áreas de maior potencial de morbimortalidade da doença em sua fase crônica, associada ao cenário de limitações de acesso aos serviços de saúde, implicando a baixa suspeição e detecção de casos crônicos e a qualidade de vida das pessoas afetadas pela doença (12). O índice de vulnerabilidade da doença de Chagas foi composto por três subíndices: indicadores epidemiológicos diretamente relacionados à doença de Chagas crônica; indicadores relacionados às doenças/agravos decorrentes da evolução da doença de Chagas; e indicadores relacionados ao acesso aos serviços de saúde (12).

### Fontes de dados

Foram utilizados dados do SIH/SUS, do Censo Demográfico de 2022 do Instituto Brasileiro de Geografia e Estatística e do índice de vulnerabilidade da doença de Chagas. 

### Viés 

A utilização de dados secundários apresentou a possibilidade de viés de registro e houve também possibilidade de subnotificação.

### Tamanho do estudo 

O estudo partiu de 25.813 registros de internações hospitalares por AVC em Minas Gerais. Os dados se referiram exclusivamente a pacientes que foram internados em leitos conveniados pelo SUS.

### Métodos estatísticos

Foram estimadas as taxas bruta e padronizada (por 100 mil habitantes) de internação hospitalar por AVC no SUS, e um mapa foi construído para apresentar tais dados. Apresentaram-se dados do índice de vulnerabilidade da doença de Chagas em Minas Gerais e por macrorregiões, em sua natureza numérica contínua. Para testar a associação entre a taxa de internação por AVC padronizada e o índice de vulnerabilidade da doença de Chagas, realizou-se o teste de correlação de Spearman. Sequencialmente, estimaram-se o número e as características dos indivíduos com internações por AVC (diagnóstico principal) e doença de Chagas/sentinelas (diagnóstico secundário). 

Os dados do SIH/SUS foram baixados no formato “.dbc”, combinados e salvos no formato “.dbf” pelo software TabWin 4.15 (14). Foram utilizados os softwares Stata, versão 17 (Statacorp, College Station, Texas, Estados Unidos) e SPSS versão 20.0 (Statistical Package for the Social Sciences, IBM) para gerenciamento do banco de dados (15), seleção de registros e análises estatísticas.

## Resultados 

Registraram-se em Minas Gerais 1.347.584 internações via SIH/SUS em 2022. Foram excluídas do estudo 1.321.771 internações, conforme os critérios de exclusão adotados (Figura 1).

**Figura 1:** Número de internações via sistema de informação hospitalar e seleção das internações de interesse, segundo critérios de inclusão e exclusão adotados neste estudo. Minas Gerais, 2022 (n=1.347.584)

Entre as internações por AVC no SUS de Minas Gerais (n=25.823), 52,2% ocorreram entre pessoas do sexo masculino, variando de 49,8% a 56,6% por macrorregião. A média de idade foi 67,4 anos, variando de 66,8 a 70,6 anos por macrorregião. A maioria se classificava com raça/cor da pele parda (43,5%) e não veio a óbito durante a internação (87,2%).

A taxa de internação por AVC encontrada em Minas Gerais foi 125,7 por 100 mil habitantes. Entre as macrorregiões do estado, Vale do Jequitinhonha, Nordeste e Norte apresentaram maiores taxas de internação por AVC, chegando a 199,^7^, 177,2 e 166,3 por 100 mil habitantes (Figura 2). Os maiores índices de vulnerabilidade da doença de Chagas foram observados nas regiões Vale do Jequitinhonha, Norte e Noroeste de Minas Gerais (Tabela 1).

**Tabela 1:** Taxa de internação por acidente vascular cerebral (AVC) calculada e índice de vulnerabilidade da doença de Chagas. Macrorregiões de Minas Gerais, 2022 (n=25.813)

Macrorregiões	n (%)	População	Taxa de internação por AVC/100 mil habitantes	Índice de vulnerabilidade da doença de Chagas
Centro	6.944 (26,9)	6.376.699	108,9	0,154
Centro Sul	1.062 (4,1)	765.591	138,7	0,214
Vale do Jequitinhonha	770 (3,0)	385.590	199,7	0,593
Leste	920 (3,6)	643.031	143,1	0,154
Leste do Sul	949 (3,7)	690.204	137,5	0,125
Nordeste	1.373 (5,3)	774.768	177,2	0,203
Noroeste	615 (2,4)	712.909	86,3	0,494
Norte	2.682 (10,4)	1.612.749	166,3	0,550
Oeste	1.632 (6,3)	1.288.118	126,7	0,190
Sudeste	2.511 (9,7)	1.596.341	157,3	0,216
Sul	3.473 (13,5)	2.796.445	124,2	0,197
Triângulo do Norte	1.169 (4,5)	1.318.243	88,7	0,333
Triângulo do Sul	766 (3,0)	787.105	97,3	0,427
Vale do Aço	947 (3,7)	790.925	119,7	0,117

**Figura 2:** Taxas de internação por acidente vascular cerebral (AVC). Macrorregiões de Minas Gerais, 2022

A correlação entre a taxa de AVC e o índice de vulnerabilidade da doença de Chagas foi fraca (r=0,145; p-valor 0,607), embora as macrorregiões Vale do Jequitinhonha e Norte, que apresentaram alto índice de vulnerabilidade da doença de Chagas, também se mostraram com alta taxa de internação por AVC (Figura 3).

**Figura 3:** Relação entre a taxa de internação por acidente vascular cerebral (AVC) por 100 mil habitantes e o índice de vulnerabilidade da doença de Chagas. Macrorregiões de Minas Gerais, 2022

Do total de internações por AVC do estado, foram observadas duas internações com registro específico da doença de Chagas como causa secundária e 16 com registros sentinelas da doença de Chagas como causa secundária (Figura 1). Entre esses 18 casos, observou-se que 12 eram mulheres, somente três indivíduos apresentavam até 60 anos, nove eram pardos, cinco morreram após a ocorrência do AVC e todos esses óbitos registrados corresponderam a pessoas do sexo feminino, e 10 dos 18 casos ocorreram na macrorregião Centro (Tabela 2).

**Tabela 2:** Descrição dos casos das internações por acidente vascular cerebral (AVC) como causa primária e doença de Chagas/sentinelas como causa secundária. Minas Gerais, 2022 (n=18)

Internações por AVC com doença de Chagas como causa secundária	CID-10^a^ primário	CID-10 secundário	Sexo	Idade	Raça/cor da pele	Cidade	Macrorregião	Morte
1°	I64	B57.2^b^	Masculino	59	Preta	Pouso Alegre	Sul	Não
2°	I64	B57^c^	Feminino	82	Preta	Uberlândia	Triângulo do Norte	Não
3°	163.9	144.2	Feminino	96	Sem informação	Belo Horizonte	Centro	Sim
4°	I64	I50.0	Feminino	83	Amarela	Belo Horizonte	Centro	Sim
5°	I64	I50.0	Masculino	52	Preta	Belo Horizonte	Centro	Não
6°	I64	I50.0	Feminino	75	Branca	Uberlândia	Triângulo do Norte	Não
7°	I63.4	I42.9	Feminino	82	Branca	Tocos do Moji	Sul	Sim
8°	I64	I50.0	Masculino	59	Parda	Sabinópolis	Centro	não
9°	I64	I50.0	Feminino	60	Parda	Sabinópolis	Centro	Não
10°	I64	I50.0	Masculino	64	Parda	Sabinópolis	Centro	Não
11°	I64	I50.0	Masculino	73	Preta	Sabinópolis	Centro	Não
12°	I64	I50.0	Feminino	62	Parda	Sabinópolis	Centro	Não
13°	I64	I49.9	Masculino	61	Branca	Pouso Alegre	Sul	Não
14°	I64	I42.9	Feminino	71	Branca	Monte Sião	Sul	Não
15°	I64	I50.0	Feminino	95	Parda	Materlândia	Centro	Não
16°	I64	I49.9	Feminino	83	Parda	Juatuba	Centro	Sim
17°	I64	I44	Feminino	78	Branca	Cambuí	Sul	Não
18°	I64	I46.1	Feminino	62	Branca	Bom Despacho	Oeste	Sim

^a^CID-10: 10ª revisão da Classificação Estatística Internacional de Doenças e Problemas Relacionados com a Saúde; ^b^CID-10 secundário específico para doença de Chagas crônica com comprometimento cardíaco; ^c^CID-10 secundário específico para doença de Chagas.

## Discussão 

Este estudo identificou alta taxa de internação por AVC pelo SUS, em 2022, em Minas Gerais. Tais taxas apresentaram considerável variação entre as macrorregiões do estado. As macrorregiões que apresentaram altas taxas de internações por AVC mostraram também os maiores índices de vulnerabilidade da doença de Chagas; contudo, a correlação entre taxa de internação por AVC e índice de vulnerabilidade da doença de Chagas foi baixa. Do total de internações por AVC registradas no estado, apenas 18 tiveram a doença de Chagas/sentinelas como causa secundária, com a maioria dos casos concentrados na macrorregião Centro. Esses achados sugerem subnotificação da doença de Chagas.

Estes dados precisam ser interpretados considerando as limitações do estudo. Tratou-se de estudo com a utilização de dados secundários, havendo sempre grau de incerteza quanto à qualidade dos registros. Embora tenha sido utilizado um banco de dados público e robusto, os registros do SIH/SUS são realizados com a finalidade principal de faturamento hospitalar (16), não sendo voltados especificamente para pesquisas, o que pode acarretar dados incertos, não padronizados, bem como a subnoticação de registros. 

Apesar de suas limitações, nos últimos anos, tem havido interesse crescente da comunidade científica pelo uso de grandes bases de dados secundárias provenientes dos serviços de saúde (17,18), devido à abrangente cobertura populacional e à oportunidade de explorar grandes amostras, com custos reduzidos (18-20). Assim, os resultados apresentados são relevantes por abranger as internações em leitos conveniados pelo SUS de todo o estado mineiro, o que agrega utilidade e interpretação a registros já existentes (18-20). 

As macrorregiões de Minas Gerais com maiores taxas de internação por AVC no SUS corresponderam aos locais com maiores índices de vulnerabilidade da doença de Chagas do Brasil, sendo elas: Vale do Jequitinhonha e Norte de Minas Gerais. Este achado corroboraria a possível relação entre AVC e doença de Chagas. No entanto, não houve nenhum registro de AVC como causa primária e doença de Chagas/sentinelas como causa secundária nas macrorregiões supracitadas, sabidamente hiperendêmicas para a doença (21,12). Esse achado foi inesperado, pois nessas regiões se encontravam a maior endemicidade para doença de Chagas (9,2%) (21). Entretanto, tais regiões também já foram reconhecidas pela precária assistência ao paciente com a doença de Chagas (22,23), onde o acesso desses pacientes a cuidados médicos adequados ainda representa importante desafio para os sistemas de saúde. A deficiência dos serviços de saúde na atenção à doença de Chagas em tais regiões pode ser uma possível explicação para o baixo número de registros, que talvez não capte a realidade (22). 

A macrorregião Centro, onde se localizava a capital do estado, congregou características de maior desenvolvimento e mostrou melhor organização dos serviços de saúde (24,25), mas não era hiperendêmica para doença de Chagas e foi a que mais apresentou notificação dos casos de AVC como causa primária e doença de Chagas/sentinelas como causa secundária a internação. Este achado reforçou a hipótese de que as características da organização dos serviços parecem interferir na qualidade dos registros médicos da internação de forma mais substancial que o próprio quadro epidemiológico. As avaliações de resolubilidade efetuadas com base no desempenho da prestação de serviços hospitalares por nível de atenção em 2010 revelaram que a macrorregião Centro apresentou resolubilidade acima de 95,0%, enquanto a macrorregião Vale do Jequitinhonha apresentou resolubilidade de 28,0%. Os valores de resolubilidade abaixo de 30,0% indicaram vazio assistencial (25).

Das internações do SUS por AVC registradas em Minas Gerais, apenas dois casos apresentaram notificação da doença de Chagas como diagnóstico secundário, enquanto outros 16 apresentaram condições sentinelas da doença de Chagas. Os achados apontaram provável subnotificação da doença de Chagas nos registros hospitalares. O próprio Ministério da Saúde afirmou subnotificação da doença de Chagas, ao apontar a prevalência de 2,1% para Minas Gerais, reconhecendo que o número de pacientes notificados foi muito aquém do real número de pessoas com a doença (26). Se houve subnotificação do diagnóstico da doença de Chagas de forma isolada, não é de se estranhar que a notificação seja ainda menor quando ofuscada por condição de emergência, como o AVC. A doença de Chagas é uma doença negligenciada (12) tanto pelos pacientes que podem não informar aos profissionais que possuem a doença, quanto pelos profissionais que não associam as complicações da doença de Chagas com a própria doença em si (22,23).

O reduzido número de registros de AVC como diagnóstico primário associado à doença de Chagas como diagnóstico secundário dificulta a adequada estimativa da magnitude da associação entre essas duas condições no contexto hospitalar público. Esperava-se número maior de registros, uma vez que há estudos na literatura que apontam a relação significativa entre a doença de Chagas e a ocorrência de AVC (4,27). Avaliaram-se 201 participantes sendo 100 controles (sem AVC) e 101 casos (com AVC). Observou-se, após realizar a sorologia para doença de Chagas, que, no grupo controle, somente dois indivíduos testaram positivo, enquanto, no grupo caso, 14 indivíduos apresentaram sorologia positiva para doença de Chagas. O resultado positivo da sorologia (OR 7,17; IC95% 1,50; 34,^19^) permaneceu independentemente associado ao AVC (27).

Foi verificada anteriormente incidência de AVC de 20,2 por 1 mil entre pessoas com doença de Chagas e de 13,9 por 1 mil entre pessoas sem doença de Chagas (p-valor 0,48). A doença de Chagas foi um preditor independente para a morte (41,6 vs. 43,1 por 1 mil entre pessoas com doença de Chagas e sem doença de Chagas) (4). Em um ambulatório de cardiologia na Bahia, o AVC isquêmico foi significativamente mais frequente em pacientes com doença de Chagas do que naqueles com cardiomiopatias embólicas não relacionadas à doença de Chagas (15,0% vs. 6,3%; p-valor 0,01) (6). Estudos patológicos do cérebro de pacientes com a forma crônica da doença de Chagas relataram frequência de infarto cerebral entre 9,4% e 36,0% (28). Além disso, o diagnóstico da doença de Chagas pode ser estabelecido após apresentação do AVC em pelo menos 40,0% dos casos (5). Foi apontado que a ocorrência de AVC poderia oportunizar o diagnóstico da doença de Chagas (27,5). Por outro lado, pacientes com doença de Chagas deveriam ser alertados sobre o risco de AVC e devidamente acompanhados (5). Todos esses estudos evidenciaram a relação entre doença de Chagas e AVC e corroboram a hipótese de que uma parcela das internações por AVC deve ser de pessoas com doença de Chagas não notificadas, especialmente nas áreas hiperendêmicas.

A taxa de internação por AVC é uma forma indireta de avaliação da disponibilidade de ações básicas de prevenção e controle da doença hipertensiva e é útil para subsidiar o planejamento, a gestão e a avaliação de políticas e ações voltadas para a atenção à saúde do adulto (29). Assim como o AVC está associado à doença hipertensiva e a outras condições de saúde (30), já foi apontado pela literatura científica que o risco de AVC é maior entre pessoas com doença de Chagas (4), porém essa associação tem sido pouco difundida. As ocorrências de AVC deveriam representar uma oportunidade de diagnóstico da doença de Chagas, contribuindo para a retirada da invisibilidade que a permeia.

Em 2022, houve elevada prevalência de internações por AVC em Minas Gerais pelo SUS, com destaque para as macrorregiões Vale do Jequitinhonha e Norte do estado, áreas caracterizadas por elevado índice de vulnerabilidade da doença de Chagas e reconhecidas como hiperendêmicas para essa infecção. No entanto, apenas 18 casos registraram AVC como causa primária e doença de Chagas/sentinelas como causa secundária. Nenhum desses registros foi identificado nas macrorregiões hiperendêmicas, sendo a maioria observada na macrorregião Centro. 

Estes achados reforçam a necessidade de sensibilizar os profissionais de saúde para a investigação sistemática da doença de Chagas em pacientes internados por AVC, a fim de aprimorar o diagnóstico, a notificação e o acompanhamento desses casos, bem como garantir maior qualidade e fidedignidade dos registros no SIH/SUS. Tal iniciativa é essencial para enfrentar o cenário de negligência histórica que ainda permeia a doença de Chagas no Brasil.
